# Oxime derivative TFOBO promotes cell death by modulating reactive oxygen species and regulating NADPH oxidase activity in myeloid leukemia

**DOI:** 10.1038/s41598-022-11543-8

**Published:** 2022-05-07

**Authors:** Ahyoung Jo, Jae-Hwan Kwak, Soo-Yeon Woo, Bo-Young Kim, Yonghae Son, Hee-Seon Choi, Jayoung Kim, Munju Kwon, Hyok-Rae Cho, Seong-Kug Eo, Ji Ho Nam, Hyung-Sik Kim, Ninib Baryawno, Dongjun Lee, Koanhoi Kim

**Affiliations:** 1 Department of Pharmacology, School of Medicine, Pusan National University, Yangsan, 50612 Republic of Korea; 2 College of Pharmacy, Kyungsung University, Busan, 48434 Republic of Korea; 3 Department of Convergence Medicine, School of Medicine, Pusan National University, Yangsan, 50612 Republic of Korea; 4 Department of Neurosurgery, College of Medicine, Kosin University, Busan, 49267 Republic of Korea; 5 College of Veterinary Medicine and Bio-Safety Research Institute, Jeonbuk National University, Iksan, 54596 Republic of Korea; 6 Department of Radiation Oncology, Pusan National University School of Medicine, Yangsan, 50612 Republic of Korea; 7 Department of Life Science in Dentistry, School of Dentistry, Pusan National University, Yangsan, 50612 Republic of Korea; 8 Childhood Cancer Research Unit, Department of Women’s and Children’s Health, Karolinska Institutet, 17177 Stockholm, Sweden

**Keywords:** Cancer, Haematological cancer

## Abstract

Several derivatives derived from the oxime structure have been reported as potential anticancer agents in various cancers. Here, we first tested a novel oxime-containing derivative of 2-((2,4,5-trifluorobenzyl)oxy)benzaldehyde oxime (TFOBO) to evaluate its anticancer effect in myeloid leukemic cells. Compared to (2-((2,4,5-trifluorobenzyl)oxy)phenyl)methanol (TFOPM), the oxime derivative TFOBO suppresses leukemic cell growth by significantly increasing reactive oxygen species (ROS) levels and cell death. Leukemic cells treated with TFOBO displayed apoptotic cell death, as indicated by nuclear condensation, DNA fragmentation, and annexin V staining. TFOBO increases Bax/Bcl2 levels, caspase9, and caspase3/7 activity and decreases mitochondrial membrane potential. ROS production was reduced by *N*-acetyl-l-cysteine, a ROS scavenger, diphenyleneiodonium chloride, a nicotinamide adenine dinucleotide phosphate (NADPH) oxidase inhibitor, after exogenous TFOBO treatment. ROS inhibitors protect leukemic cells from TFOBO-induced cell death. Thus, our study findings suggest that TFOBO promotes apoptosis by modulating ROS and regulating NADPH oxidase activity. Collectively, the oxime-containing derivative TFOBO is a novel therapeutic drug for myeloid leukemia.

## Introduction

Acute myeloid leukemia (AML) is a blood cancer caused by the rapid proliferation of abnormal myeloid blasts in the blood, bone marrow, and other tissues^1^, making it difficult for normal blood cells to perform their work. AML features symptoms such as fever, fatigue, and hemorrhage^2^. In the United States, there were an estimated 20,240 new AML cases and 11,400 AML-related deaths in 2021. AML is the most frequent form of leukemia and it is diagnosed most often in 65- to 74-year-old people. Chemotherapy is the mainstay of treatment, and the survival rate of patients has increased with advances in treatment^3^. However, despite considerable research and development, it is difficult to induce remission, and the risk of relapse is high because of chemotherapy resistance^2^. Accordingly, novel therapeutic approaches with low-risk and outstanding effects are urgently needed.

Cell death, which maintains an adequate cell count, is generally classified into two types: accidental cell death and regulated cell death^4^. Regulated cell death is classified as necrosis, autophagy, ferroptosis, pyroptosis, and apoptosis^5^. Necrosis is a passive cell death that destroys cell membranes and cell structures in response to changes in the surrounding environment, such as nutrient deficiency and mechanical shock^6^. Autophagy is the highly conserved catabolic process involving the formation of double-membrane vesicles called autophagosomes^7^. Ferroptosis is an iron-dependent form of regulated cell death caused by unrestricted lipid peroxidation and subsequent membrane damage^8^. Pyroptosis is a form of lytic cell death that is triggered by pro-inflammatory signals and associated with inflammation^9^. Apoptosis is an active process of programmed cell death that eliminates unnecessary or damaged cells and is essential for the survival of complex organisms^10^. Thus, the inhibition of apoptosis is linked to a variety of cancers and diseases such as atherosclerosis. In contrast, an increase in apoptosis is associated with diseases such as AIDS and Alzheimer’s disease^6^. Apoptosis has cellular morphological features, such as nuclear DNA fragmentation, cell shrinkage, and chromatin condensation^11^. There are two main mechanisms of apoptosis: an extrinsic pathway and an intrinsic pathway. In the extrinsic pathway, a specific ligand binds to the death receptor present on the cell membrane, which activates caspase8 and caspase10, the initiator caspase, and induces apoptosis^12^. The intrinsic pathway, a mitochondria-dependent pathway, is associated with the Bcl2 family. Anti-apoptotic members of the Bcl2 family, such as Bcl2, discontinue releasing cytochrome C^13^. Pro-apoptotic members, such as Bax, induce mitochondrial outer membrane permeabilization, allowing the release of specific proteins such as cytochrome C into the cytoplasm^14^. These pro-apoptotic factors form an apoptosome, which activates caspase9. Caspase9 cleaves and activates effector caspases such as caspase3 and caspase7^12^. Activated caspase3 cleaves poly(ADP-ribose) polymerase and causes membrane blebbing and DNA fragmentation cleavage^15^. This eventually leads to apoptosis.

In this study, we first tested the anticancer effects of novel oxime-containing derivatives 2-((2,4,5-trifluorobenzyl)oxy)benzaldehyde oxime (TFOBO) and (2-((2,4,5-trifluorobenzyl)oxy)phenyl)methanol (TFOPM) in myeloid leukemic cells. Here we report that exogenous TFOBO treatment suppresses leukemic cell growth by significantly increasing reactive oxygen species (ROS) levels and cell death. In addition, ROS production was reduced by *N*-acetyl-l-cysteine (NAC), a ROS scavenger, or diphenyleneiodonium chloride (DPI), a nicotinamide adenine dinucleotide phosphate (NADPH) oxidase inhibitor, after exogenous TFOBO treatment. Collectively, our study findings suggest that TFOBO promotes apoptosis by modulating ROS and regulating NADPH oxidase activity, making it a novel therapeutic drug for myeloid leukemia.

## Materials and methods

### Cell culture and treatments

Human acute monocytic leukemia THP1 cells were purchased from the American Type Culture Collection. The THP1 cells were cultured in RPMI1640 medium supplemented with 10% fetal bovine serum (FBS) and 1% penicillin–streptomycin in a humidified atmosphere of 5% CO_2_ at 37 °C. After the THP1 cells (2.5 × 10^5^ cells/ml) were serum-starved overnight in an RPMI medium containing 0.1% bovine serum albumin (BSA; GenDEPOT), the cells were treated with control vehicle, TFOBO, and TFOPM.

### Chemical and reagent

**Figure 1:**
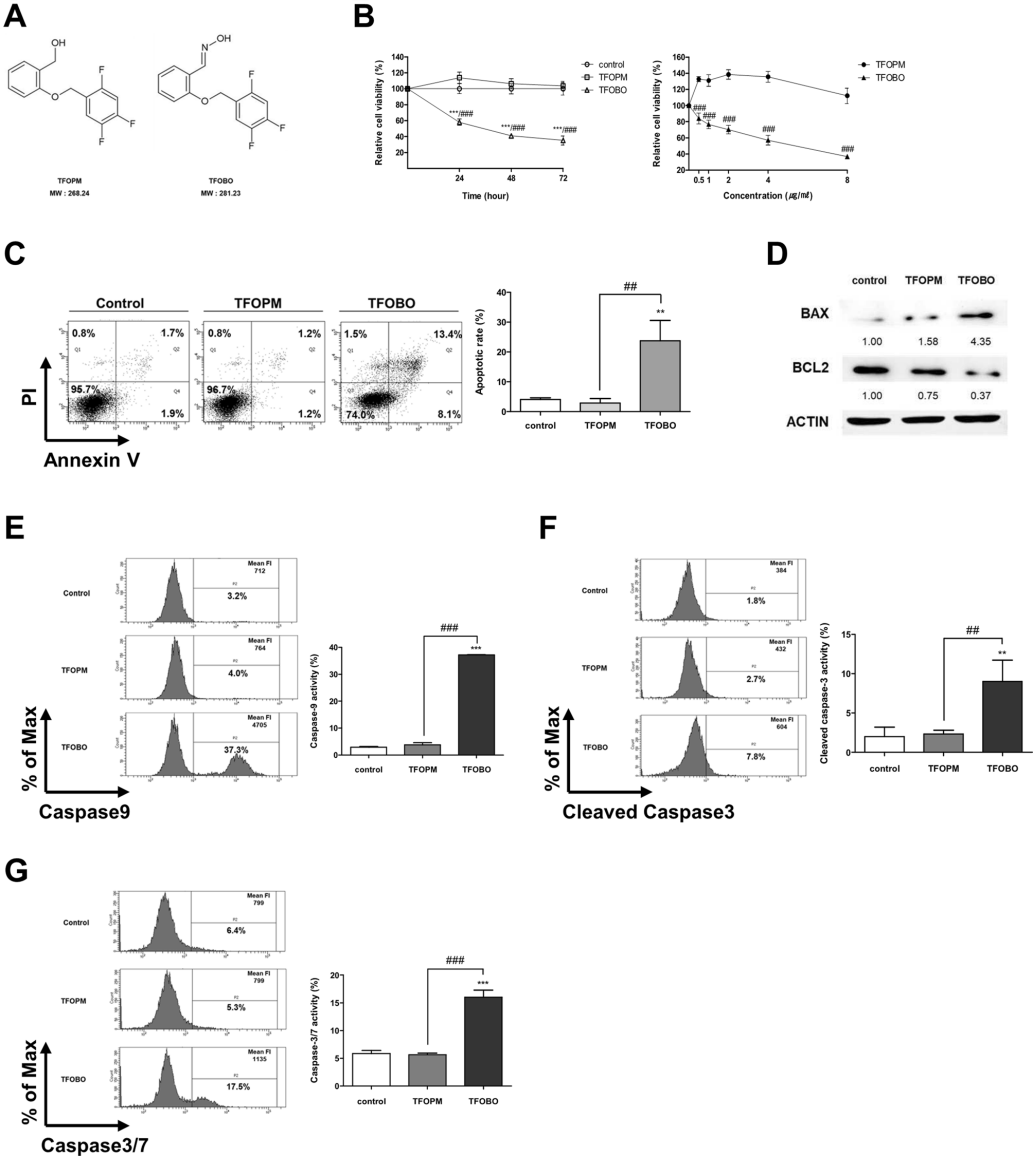
Exogenous addition of ((2,4,5-trifluorobenzyl)oxy)-benzene derivative (TFOBO) affects myeloid leukemic cell growth. (**A**) Chemical structure of TFOPM and TFOBO. (**B**) Serum-starved THP1 cells are treated with 4 µg/ml of TFOBO for 24, 48, and 72 h (left) and then treated with indicated concentrations of TFOBO for 48 h (right). Cell viability is measured using a CCK-8 assay. (***p < 0.001 vs. control; ^###^p < 0.001 vs. (2-((2,4,5-trifluorobenzyl)oxy)phenyl)methanol [TFOPM]). (**C**) THP1 cells are serum-starved overnight and treated with 4 µg/ml of TFOBO for 48 h. The cells are double-stained with annexin V-APC and propidium iodide and analyzed by flow cytometry. The apoptotic cell percentage data are shown in the graph. (**p < 0.01 vs. control; ^##^p < 0.01 vs. TFOPM). (**D**) After 48 h incubation with 4 µg/ml of TFOBO, the THP-1 cells are lysed. After quantifying the protein concentration of cell lysate by bicinchoninic acid assay, equal amounts of proteins (20 µg) are separated by sodium dodecyl sulphate–polyacrylamide gel electrophoresis and transferred to the nitrocellulose membrane. The membrane is probed with anti-BAX and anti-BCL-2 antibodies. β-ACTIN is used as a loading control. The representative data of the western blot analysis are shown. (**E–G**) After serum starvation overnight and treatment with 4 µg/ml of TFOBO for 48 h, the caspase activity of THP1 cells is measured by flow cytometry. (**E**) FITC-LEHD-FMK is added to the cells, which are then incubated at 37 °C for 1 h, and the caspase9 activity is observed. (**F**) The cells are stained with cleaved caspase3 antibody after fixation and permeabilization and their fluorescence is measured. (**G**) For the detection of caspase-3/7 activity, the cells are treated with TF2-DEVD-FMK for 3 h and analyzed. (**p < 0.01, ***p < 0.0001 vs. control; ^##^p < 0.01, ^###^p < 0.0001 vs. TFOPM). The data in the graphs are presented as mean ± SD (n = 3 replicates for each group). Representative data from three independent experiments are shown.

Antibodies against β-actin, anti-mouse immunoglobulin G horseradish peroxidase (IgG-HRP), and anti-rabbit IgG-HRP were purchased from Santa Cruz Biotechnology, Inc. Anti-BAX and anti-BCL2 antibodies were obtained from Cell Signaling Technology, Inc. The substituted ((2,4,5-trifluorobenzyl)oxy)-benzene derivatives were provided by Professor Jae-Hwan Kwak (College of Pharmacy, Kyungsung University). The chemical structures of the (2,4,5-trifluorobenzyl)oxy)-benzene derivatives are shown in Fig. 1A. The derivatives were dissolved in dimethyl sulfoxide to produce a 4 g/ml stock solution and stored at − 20 °C. The stock solution was diluted in a cell culture medium as required.

### Cell viability assay

Cell viability was measured using a Vi-Cell cell counter (Beckman Coulter, Inc.) and a cell counting kit 8 assays (CCK8; Dojindo). To evaluate cell viability using the CCK8 assay, the THP1 cells were seeded into 96-well plates and treated with 2-substituted-((2,4,5-trifluorobenzyl)oxy)-benzene derivatives. After treatment, 10 µl of CCK8 reagent was added to each well and incubated for 3 h at 37 °C using the CCK8 kit protocol. The absorbance differences were measured at 450 nm wavelength.

### Annexin V/PI apoptosis assay

An apoptosis assay was conducted using the Annexin V-APC apoptosis detection kit (Life Technologies Corporation) according to the manufacturer’s instructions. After 48 h of treatment with 4 µg/ml ((2,4,5-trifluorobenzyl)oxy)-benzene derivatives, THP1 cells were harvested. The cells were washed with phosphate-buffered saline (PBS) and resuspended in 100 µl of binding buffer. Five microliters of annexin V-APC were added and incubated for 15 min at room temperature in dark. After washing with binding buffer, the cells were resuspended in a 500 µl binding buffer. Five microlitres of propidium iodide were added and the cells were analyzed by FACS Canto 2 flow cytometry (BD Biosciences).

### Caspase activity assay

Caspase9 activity was observed using the caspase9 FITC staining kit (Abcam) according to the manufacturer’s protocol. THP1 cells (10^6^ cells/ml) were added to FITC-LEHD-FMK as a fluorescent marker and incubated at 37 °C and 5% CO_2_ for 1 h. The cells were washed twice with washing buffer and the caspase-9 activity was subjected to flow cytometric analysis. Cleaved caspase3 activity was determined by flow cytometry using a caspase3 antibody (R&D Systems). THP1 cells were harvested and washed twice with PBS. The cells were then fixed with 4% paraformaldehyde and incubated at room temperature for 10 min. After being washed twice with PBS, the cells were permeabilized with ice-cold 90% methanol and incubated at 4 °C for 30 min. After two washes with PBS, a caspase3 antibody (5 µl/10^6^ cells) was added and the cells were incubated at room temperature for 30 min in the dark. The cells were washed with PBS before being analyzed through flow cytometry analysis. Caspase3 and caspase7 activities of the cells were measured using a caspase3/7 activity apoptosis assay kit (AAT Bioquest) according to the manufacturer’s protocol. The THP1 cells were treated with TF2-DEVD-FMK, a fluorogenic indicator of caspase3/7 activity, and incubated at 37 °C and 5% CO_2_ for 3 h. Next, the cells were washed twice with assay buffer, and caspase3/7 activity was measured by flow cytometry.

### Measurement of intracellular ROS

Intracellular ROS production was detected using a ROS detection kit (PromoCell GmbH) according to the manufacturer’s instructions. The THP1 cells were serum-starved overnight with 0.1% BSA. The cells were then centrifuged (1000 rpm, 5 min) and resuspended in the assay buffer. The cells were seeded in 48-well plates (1.25 × 10^5^ cells/well) and treated with the ROS label 2′,7′-dichlorofluorescein diacetate (H_2_DCFDA) for 30 min at 37 °C in the dark. The medium was removed and ((2,4,5-trifluorobenzyl)oxy)-benzene derivatives were treated in assay buffer supplemented with 10% FBS for 48 h. Cells were treated with ROS inducer for 1 h before flow cytometric analysis.

### Western blot analysis

Proteins were extracted using PRO-PREP protein extraction solution (iNtRON Biotechnology). The concentration of the protein samples was determined using the BCA assay. The proteins were separated on 12% SDS-PAGE gels and transferred to nitrocellulose membranes. After blocking with 1% skim milk in Tris-buffered saline (TBS) containing 0.05% Tween-20 for 1 h at room temperature, the membranes were incubated with primary antibodies diluted in the blocking solution (1:1000) at 4 °C overnight. The membranes were washed three times with washing buffer (TBS with 0.05% Tween-20) for 15 min each and then incubated for 1 h with HRP-conjugated secondary antibodies diluted in the blocking solution (1:5000) at room temperature. After washing three times with washing buffer for 15 min each, bands were detected using chemiluminescent detection reagents (Luminata Forte Western HRP Substrate). Chemiluminescence was imaged using an Amersham Imager 680 (GE Healthcare).

### Nuclear staining

To detect nuclear morphological changes, THP1 cells were visualized using fluorescence confocal microscopy. THP1 cells were treated with 4 µg/ml of ((2,4,5-trifluorobenzyl)oxy)-benzene derivatives for 48 h and fixed with 4% paraformaldehyde in phosphate-buffered saline (PBS) for 10 min at room temperature. The fixed cells were washed with PBS and stained with DAPI solution for 10 min at room temperature. The cells were washed twice with PBS and imaged using a confocal laser scanning microscope (Olympus FV1000; Olympus).

### DNA fragmentation assay

After 48 h of treatment with 4 µg/ml of ((2,4,5-trifluorobenzyl)oxy)- benzene derivatives, cells were lysed in DNA extraction buffer containing 20 mM Tris–HCl (pH 7.4), 100 mM NaCl, 5 mM EDTA (pH 8.0) and 0.5% SDS for 30 min at room temperature. Then, proteinase K was treated at 200 µg/ml, and samples were incubated for 3 h at 55 °C. Extraction of DNA was added with an equal volume of phenol: chloroform: isoamyl alcohol (25:24:1, v/v/v) and centrifuged (15,000 rpm, 20 min, 4 °C). The aqueous phase was incubated with DNase-free RNase for 1 h at 37 °C and the DNA was extracted by phenol: chloroform: isoamyl alcohol. The supernatant was added with 5 M NaCl and isopropanol and incubated at − 20 °C overnight. After centrifugation at 15,000 rpm for 20 min at 4 °C, the pellets were washed with 70% ethanol, and the dried pellets were dissolved in TE buffer (10 mM Tris–HCl and 1 mM EDTA). The DNA samples (10 µg) were separated on 1.5% agarose gel at 50 V, and the gel was stained with ethidium bromide (EtBr) and photographed on the Gel-Doc XR + Imager (Bio-Rad, Hercules, CA).

### Measurement of mitochondrial membrane potential (ΔΨm)

The fluorescent probe 5,5′,6,6′-tetrachloro-1,1′,3,3′-tetraethyl-benzimidazolylcarbocyanine iodide (JC1; Life Technologies Corporation) was used to measure mitochondrial membrane potential (MMP). After treatment with (2,4,5-trifluorobenzyl)oxy)-benzene derivatives for 48 h, the cells were stained with 5 µg/ml JC1 for 10 min at 37 °C. The cells were then washed with PBS and analyzed by flow cytometry.

### Statistical analysis

Results are expressed as mean ± standard deviation (SD) and were analyzed using one-way analysis of variance (ANOVA), followed by Tukey's multiple comparison tests and two-way ANOVA using PRISM (version 5.0; GraphPad Software Inc., San Diego, CA, USA). Statistical significance was set at p < 0.05.

## Results

### Exogenous TFOBO treatment induces the death of myeloid leukemic cells

Oxime is a chemical compound used as an antidote to nerve agents^16,17^. Several derivatives derived from the oxime structure have been reported as potential anticancer agents in various cancers^18–20^. However, the role of oxime derivatives in leukemia remains unknown. In this study, we first tested two new compounds that have structural similarities with the difference in the oxime structure as a functional group, such as 2-((2,4,5-trifluorobenzyl)oxy)-benzene derivatives, TFOBO and 2-((2,4,5-trifluorobenzyl)oxy)phenyl) methanol (TFOPM) (Fig. 1A) to evaluate their anticancer effect in myeloid leukemic cells. To assess whether oxime derivatives affect myeloid leukemic cells, we exposed THP1 myeloid leukemic cells to TFOPM and TFOBO (Fig. 1B–G). Interestingly, our analyses revealed that exogenous TFOBO treatment decreased THP1 myeloid leukemic cell growth (Fig. 1B and Supplementary Fig. S1A), and an increase in the number of apoptotic (Annexin V^+^) cells (Fig. 1C), cell shrinkage, nuclear condensation (Supplementary Fig. S1B), and DNA fragmentation (Supplementary Fig. S1C). Subsequently, we further checked BCL2 and BCL-2-associated X protein (BAX) expression after TFOBO treatment (Fig. 1D). BAX expression was indeed augmented in THP1 myeloid leukemic cells after TFOBO treatment. Interestingly, BCL2 expression was decreased in THP1 myeloid leukemic cells after TFOBO treatment. In addition, the activation of caspases plays a central role in the induction of apoptosis^21^. To determine whether TFOBO-mediated apoptosis is associated with caspase activation in THP1 myeloid leukemic cells, we measured caspase activity by flow cytometry after TFOBO treatment in THP1 myeloid leukemic cells (Fig. 1E–G). Caspase9 (Fig. 1E), cleaved caspase3 (Fig. 1F), and caspase3/7 (Fig. 1G) were highly increased in THP1 myeloid leukemic cells after TFOBO treatment.

### Sequential augmentation of ROS and apoptosis is responsible for the depletion of myeloid leukemic cells post-TFOBO treatment

**Figure 2:**
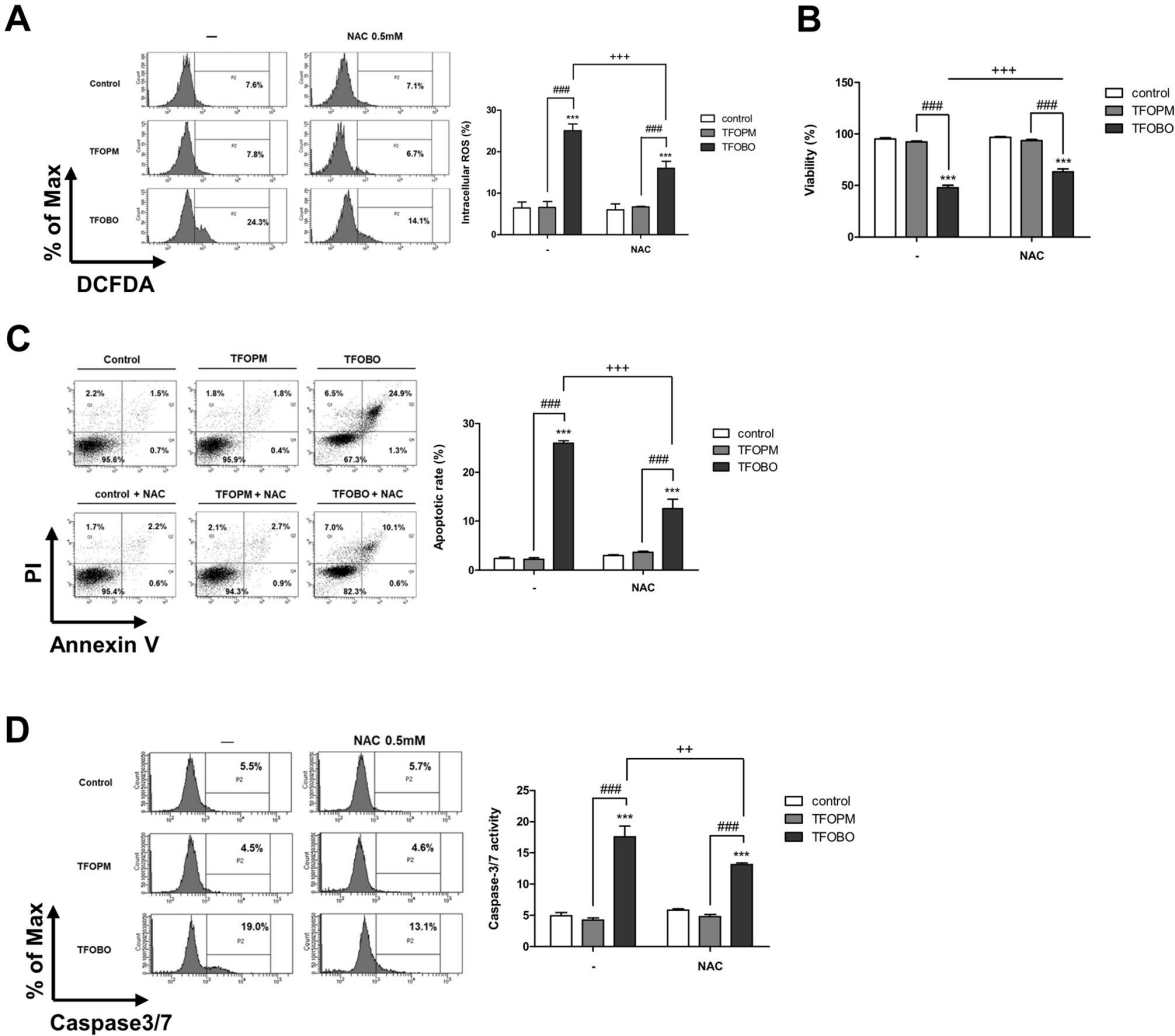
*N*-acetyl-l-cysteine (NAC) treatment rescues 2-((2,4,5-trifluorobenzyl)oxy)benzaldehyde oxime (TFOBO)–mediated cell death in myeloid leukemic cells. (**A**) Serum-starved THP1 cells are incubated with 2′,7′-dichlorofluorescein diacetate for 30 min and pre-treated with 0.5 mM NAC for 30 min before treatment with TFOBO derivatives. The reactive oxygen species generation is analysed by flow cytometric analysis, and the data are representative of three independent experiments. ***p < 0.001 vs. control; ^###^p < 0.001 vs. TFOPM; ^+++^p < 0.001 vs. TFOBO alone. (**B**) After THP1 cells are pre-treated with 0.5 mM NAC for 30 min, the cells are treated with TFOBO at 4 µg/ml for 48 h. Cell viability is measured using a Vi-cell counter. ***p < 0.001 vs. control; ^###^p < 0.001 vs. (2-((2,4,5-trifluorobenzyl)oxy)phenyl)methanol [TFOPM]; ^+++^p < 0.001 vs. TFOBO alone. (**C**) Analysis of apoptosis using annexin V and (**D**) caspase3/7 activity is observed by flow cytometry. ***p < 0.001 vs. control; ^###^p < 0.001 vs. TFOPM; ^+++^p < 0.001 vs. TFOBO alone. The graphs are presented as mean ± SD (n = 3 replicates for each group). Representative data from three independent experiments are shown.

Apoptosis is highly associated with ROS^22^. Thus, to investigate whether treatment with TFOBO is related to the generation of intracellular ROS, we assessed ROS activity after TFOBO treatment by measuring H_2_DCFDA in THP1 myeloid leukemic cells (Fig. 2A and Supplementary Fig. S2A). Our results revealed that TFOBO treatment increased ROS levels in THP1 myeloid leukemic cells. In addition, mitochondrial membrane potential (MMP) changes in apoptosis^23^. Dysfunctional mitochondria, in turn, increased ROS generation^24^. Thus, we tested MMP activity using JC1 dye through flow cytometry (Supplementary Fig. S2B). JC1 dye is a membrane-permeable lipophilic dye and exhibits red fluorescence as J-aggregates in the mitochondrial matrix in healthy cells^25–27^. When apoptosis occurs, the mitochondrial membrane is depolarized and JC1 remains as a monomer with green fluorescence in the cytoplasm. The loss of MMP was increased after TFOBO treatment. These results indicated that TFOBO could induce mitochondrial dysfunction and alter mitochondrial membranes in THP1 myeloid leukemic cells.

### Effects of NAC on TFOBO-induced apoptosis of THP1 myeloid leukemic cells

To test whether ROS level is rescued by NAC, an antioxidant that blocks ROS^28^, we pre-treated THP1 myeloid leukemic cells with 0.5 mM NAC for 30 min, followed by treatment with TFOBO for 48 h (Fig. 2A). NAC treatment rescued ROS levels (Fig. 2A), myeloid leukemic cell growth (Fig. 2B), apoptotic (Annexin V^+^) cells (Fig. 2C), caspase3/7 activation (Fig. 2D), and membrane potential (Supplementary Fig. S2C) in THP1 myeloid leukemic cells.

### Effects of DPI on TFOBO-induced ROS generation in THP1 myeloid leukemic cells

**Figure 3:**
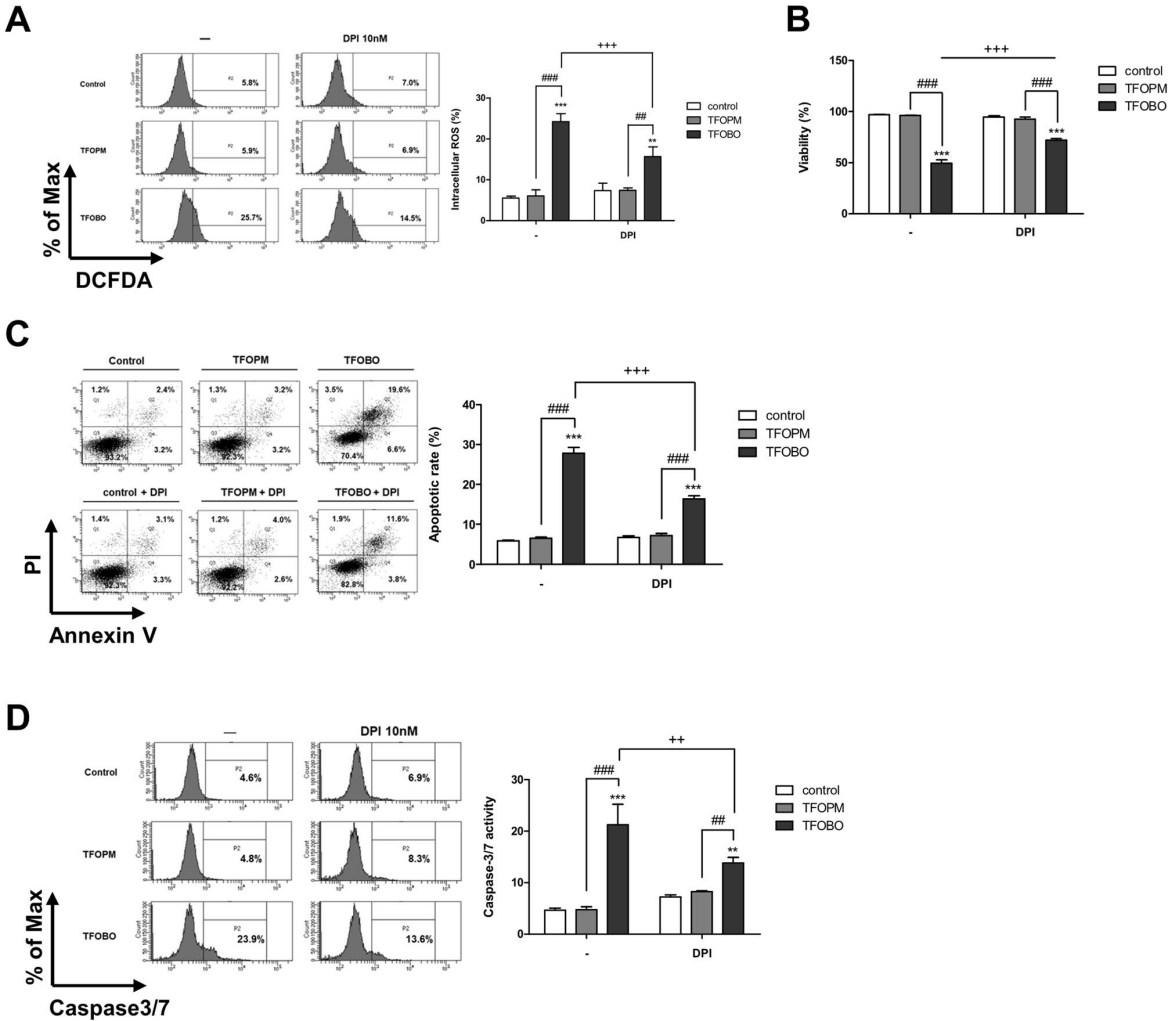
DPI treatment rescues ((2,4,5-trifluorobenzyl)oxy)-benzene derivative (TFOBO)-mediated cell death in myeloid leukemic cells. (**A)** Serum-starved THP1 cells are incubated with 2′,7′-dichlorofluorescein diacetate for 30 min and pre-incubated in 10 nM DPI for 30 min prior to treatment of TFOBO. The ROS generation is measured by flow cytometric analysis, and the representative data from three independent experiments are shown. **p < 0.01 ***p < 0.001 vs. control; ^##^p < 0.01, ^###^p < 0.001 vs. (2-((2,4,5-trifluorobenzyl)oxy)phenyl)methanol [TFOPM]; ^+++^p < 0.001 vs. TFOBO alone. (**B**) After serum-starvation, THP1 cells are pre-treated with 10 nM DPI for 30 min, followed by treatment with TFOBO at 4 µg/ml for 48 h. The viability is measured using a Vi-cell counter. (**C**) Analysis of apoptosis using annexin V and (**D**) caspase3/7 activity is subjected to flow cytometric analysis. **p < 0.01, ***p < 0.001 vs. control; ^##^p < 0.01, ^###^p < 0.001 vs. TFOPM; ^++^p < 0.01, ^+++^p < 0.001 vs. TFOBO alone. The graphs are presented as mean ± SD (n = 3 replicates for each group). Representative data from three independent experiments are shown.

NADPH oxidase is a major source of cellular ROS, produces ROS, and induces apoptosis in cancer cells^29,30^. Thus, to investigate whether NADPH oxidase is involved in TFOBO-induced ROS generation, cells were pre-treated with 10 nM DPI, an inhibitor of NADPH oxidase, for 30 min before treatment with oxime derivatives for 48 h. DPI treatment rescued ROS levels (Fig. 3A), myeloid leukemic cell growth (Fig. 3B), apoptotic (Annexin V^+^) cells (Fig. 3C), caspase3/7 activation (Fig. 3D), and membrane potential (Supplementary Fig. S2D) in THP1 myeloid leukemic cells. These results indicate that the source of TFOBO-induced ROS generation is significantly related to NADPH oxidase, which is involved in inducing apoptosis in THP1 myeloid leukemic cells.

## Discussion

**Figure 4:**
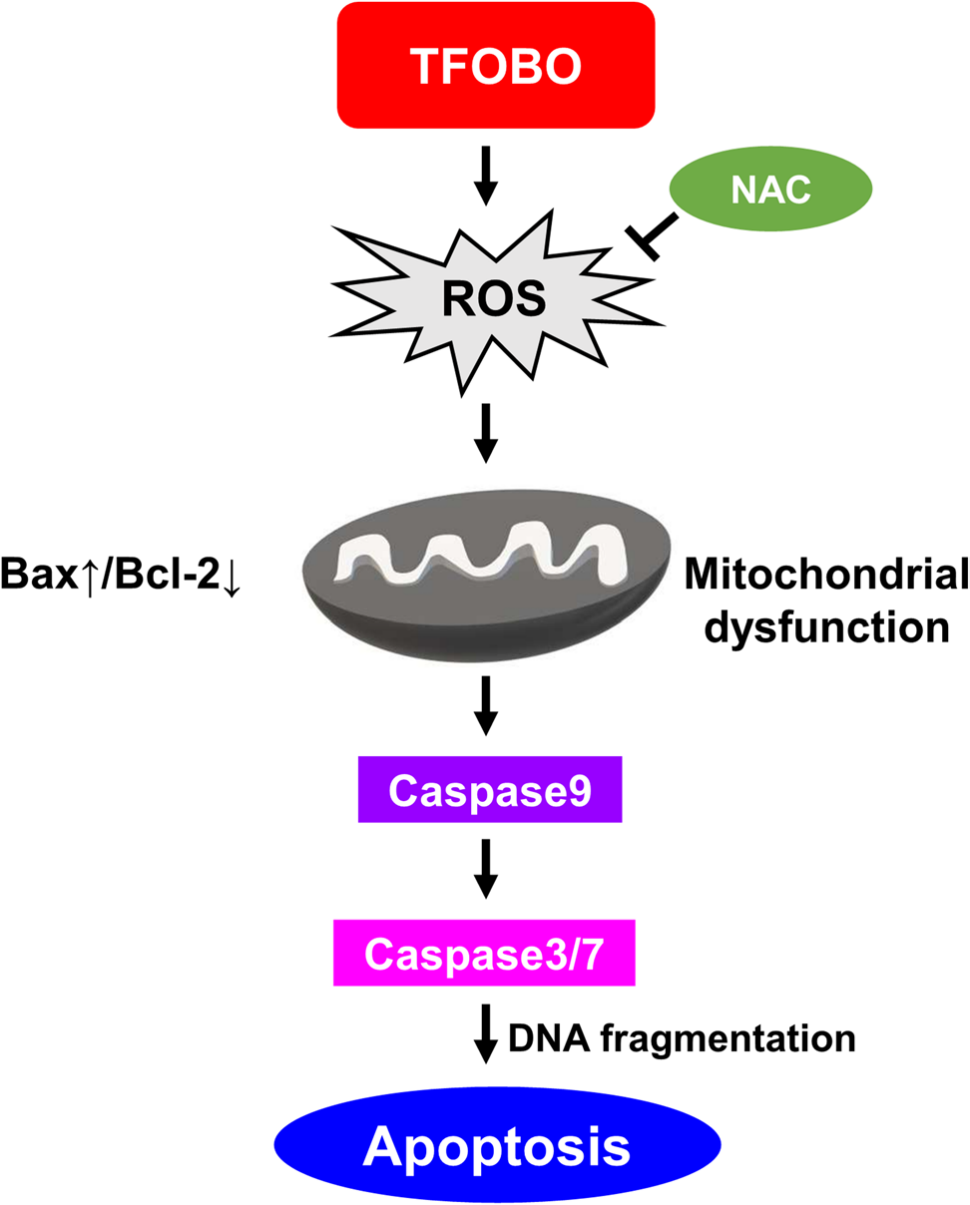
The mechanism for TFOBO-induced apoptosis in THP1 cells via ROS-mediated intrinsic pathway.

This study was the first to reveal a new pharmacological activity of oxime derivatives, contributing to its anti-cancer effects. We first demonstrated that exogenous TFOBO treatment suppressed leukemic cell growth owing to significantly increased ROS levels and cell death (Fig. 4). In addition, ROS production was reduced by NAC, a ROS scavenger, or DPI, an NADPH oxidase inhibitor, after exogenous TFOBO treatment.

AML is a malignant hematopoietic tumor and the most common form of acute leukemia in adults. Despite significant progress in treatment, it is difficult to completely cure AML and it has a high probability of recurrence due to chemical resistance^31,32^. Therefore, it is necessary to develop new more effective therapeutic agents to overcome these defects. In many studies, various compounds derived from the oxime structure have shown anti-cancer activity in several cancer cell lines^33–36^, suggesting that the oxime structure may play a major role in inducing apoptosis. Therefore, in this study, to confirm the potential of the oxime structure as a novel chemotherapeutic agent for leukemia, we investigated the mechanism of the anti-cancer activity of TFOBO, an unprecedented oxime-containing derivative, versus that of TFOPM, which is structurally similar to TFOBO.

Next, to determine whether the BCL2 family is involved in the apoptotic process induced by TFOBO in THP1 myeloid leukemic cells, we treated THP1 myeloid leukemic cells with TFOBO. The BCL2 family is reportedly a major regulator of apoptosis. The BCL2 family is divided into anti-apoptotic proteins that promote cell survival, such as BCL2 and BCL-xL, and pro-apoptotic proteins that promote cell death, such as BAX and BAK^37^. Several studies have reported that the ratio between these two groups helps determine whether apoptosis is caused by apoptotic stimulation^38^ and is a major regulator of apoptosis^39^. TFOBO treatment increased the expression level of pro-apoptotic BAX and decreased the expression level of anti-apoptotic BCL2 versus control. In response to apoptotic stimulation, BCL2 family proteins translocate to the mitochondria, cause mitochondrial damage^40^, and sequentially activate caspase9, caspase3, and caspase7^21^.

Oxidative phosphorylation is performed by the electron transport chain (ETC), and electron transport through complexes of ETC can generate superoxide and hydrogen peroxide^41,42^. ROS, including hydroxyl radicals (OH^**·**^), superoxide anions (O_2_^−**·**^), and hydrogen peroxide (H_2_O_2_), occur owing to incomplete reduction of molecular oxygen^43^ and are generated by several sources, including mitochondria, NADPH oxidases, cyclooxygenases, lipoxygenases, xanthine oxidases, and cytochrome P450 enzymes^44^. The main contributors to cellular ROS are mitochondria and NADPH oxidases^45^. In the mitochondria, ROS are produced by adenosine triphosphate synthesis through oxidative phosphorylation^46^. NADPH oxidase reduces molecular oxygen to superoxide anions and hydrogen peroxide^47^, and ROS produced by NADPH oxidase plays an important role in regulating cellular signals and killing microorganisms^48^. ROS are by-products formed during incomplete oxygen reduction and play an important role in a variety of biological activities, including intracellular signaling^49^. Immoderately high levels of ROS can trigger severe damage to cellular function, leading to a variety of diseases, including cancer and cellular aging^50^, and suggesting that excessive ROS can play a major role as a mediator in several apoptotic signaling pathways. The proof that apoptosis can be induced by ROS is provided by studies in which apoptosis was inhibited by the addition of antioxidants^51–54^. In addition, ROS generated by NADPH oxidase is presumed to induce apoptosis in cancer cells^10,12,16,29,30^. As such, ROS plays a major role in regulating biological activities, including cellular proliferation, differentiation, and signaling in most organisms^55^, but excessively elevated ROS levels that are not eliminated by antioxidant enzymes and antioxidants can have detrimental effects on nucleic acids, proteins, and lipids, leading to cellular aging and death^56^. ROS can contribute to low-level tumourigenesis in cancer cells, but since they promote high-level cell death and severe cellular damage, various drugs that affect ROS metabolism have been used as effective cancer therapy^57^.

Thus, our study findings suggest that TFOBO with an oxime structure promotes apoptosis by modulating ROS and regulating NADPH oxidase, unlike TFOPM without an oxime structure. TFOBO-induced intracellular ROS generation may be significantly involved in NADPH oxidase, and ROS plays a key role in TFOBO-induced apoptosis in THP1 myeloid leukemic cells. Collectively, TFOBO and its oxime structure may be promising molecules in the chemoprevention and chemotherapy treatment of myeloid leukemia. Thus, further investigations are needed to confirm its potential usefulness as a chemotherapeutic agent.

## Supplementary Information

Supplementary Figures.
