# Supplementary material for: Oxime derivative TFOBO promotes cell death by modulating reactive oxygen species and regulating NADPH oxidase activity in myeloid leukemia
Source: Sci Rep. 2022 May 7;12:7519. doi: 10.1038/s41598-022-11543-8 (PMC9079095; doi:10.1038/s41598-022-11543-8)

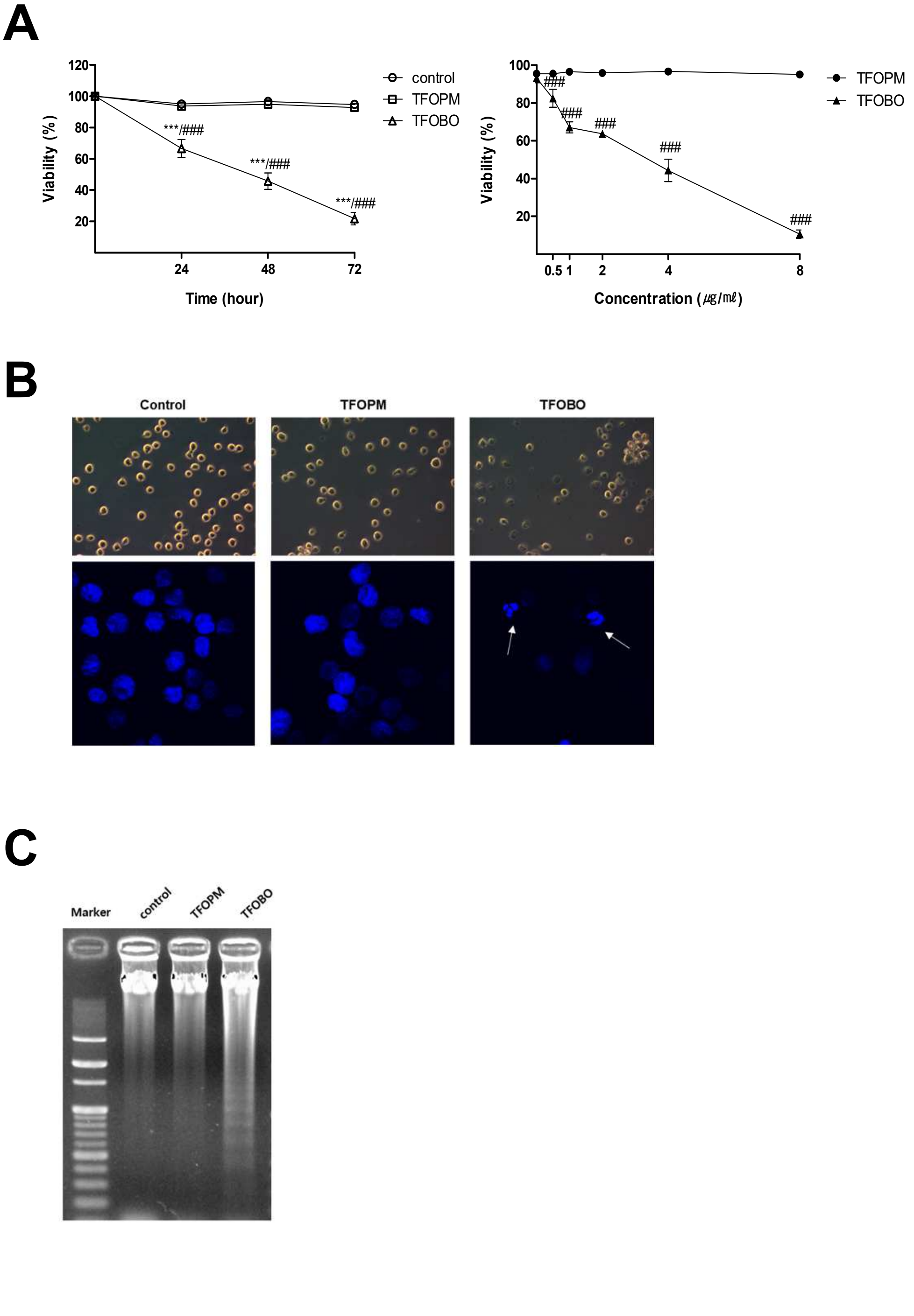

Supplemental Figure 1

Supplemental Figure 1. Exogenous addition of ((2,4,5-trifluorobenzyl)oxy)-benzene derivative (TFOBO) increases the death of myeloid leukemic cells. (A) Serum-starved THP1 cells were treated with 4 ㎍/㎖ of ((2,4,5-trifluorobenzyl)oxy)-benzene derivatives for 24, 48, and 72 h (left), and were treated with indicated concentrations of ((2,4,5-trifluorobenzyl)oxy)-benzene derivatives for 48 h (right). Cell viability was measured using a Vi-cell counter. (***$p < 0.001$, vs. control; ###$p < 0.001$, vs. TFOPM). The graphs are presented as the mean ± SD (n = 3 replicates for each group). The representative data from three independent experiments are shown. **(B)** Effects of ((2,4,5-trifluorobenzyl)oxy)-benzene derivatives on morphological changes in THP1 cells. After treatment with 4 ㎍/㎖ of ((2,4,5-trifluorobenzyl)oxy)-benzene derivatives for 48 h, morphological changes in THP1 cells were visualized with a light microscope. To measure nuclear morphological changes, the cells were fixed and stained with DAPI solution for 10 min. The stained cells were observed with the confocal microscope. **(C)** Effects of ((2,4,5-trifluorobenzyl)oxy)-benzene derivatives on DNA fragmentation in THP1 cells. Serum-starved THP1 cells (2.5×10^5 cells /㎖) were treated with 4 ㎍/㎖ of ((2,4,5-trifluorobenzyl)oxy)-benzene derivatives for 48 h. Extraction of DNA was analyzed on a 1.5% agarose gel and stained with EtBr. Marker indicates the 100 bp ladder is used as a molecular size marker.

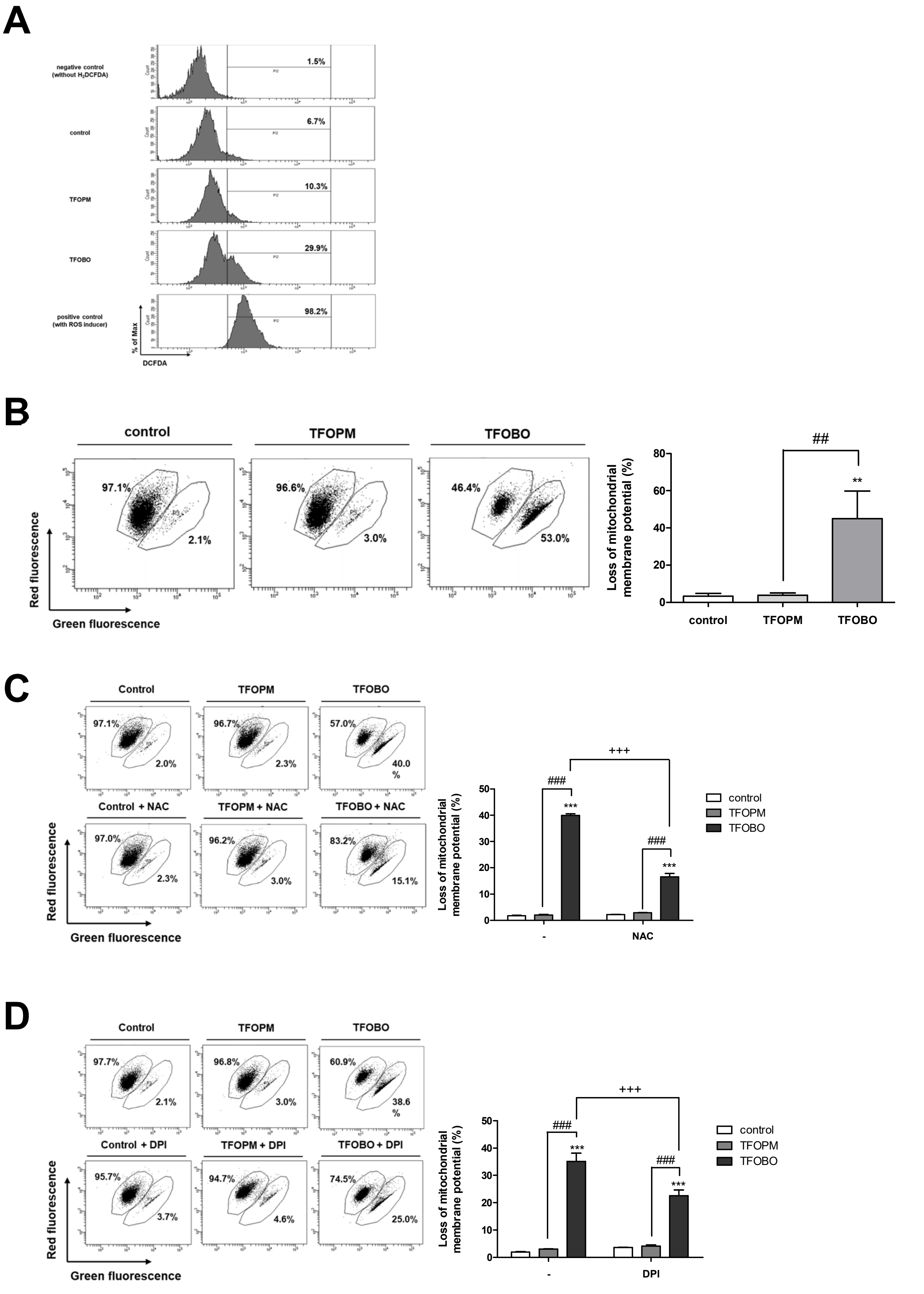

Supplemental Figure 2

Supplemental Figure 2. Exogenous addition of ((2,4,5-trifluorobenzyl)oxy)-benzene derivative (TFOBO) affects ROS and mitochondrial membrane potential (MMP) in THP1 cells. (A) After serum-starvation, THP1 cells were incubated with H2DCFDA for 30 min, and ((2,4,5-trifluorobenzyl)oxy)-benzene derivatives were added at 4 ㎍/㎖ to the cells for 48 h. The ROS inducer was treated to the cells for 1 h before analysis. The level of intracellular ROS generation was detected by flow cytometry. **(B)** Effects of ((2,4,5-trifluorobenzyl)oxy)-benzene derivatives on mitochondrial membrane potential (MMP) in THP1 cells. Serum-starved THP1 cells were treated with 4 ㎍/㎖ of ((2,4,5-trifluorobenzyl)oxy)-benzene derivatives for 48 h and stained with JC1 dye at 37 ℃ for 10 min. The percentage of green fluorescence due to depolarized MMP was analyzed by flow cytometry. The percentages of depolarized MMP are shown in the graph. **p < 0.01, vs. control; ##p < 0.01, vs. TFOPM. **(C)** After THP1 cells were pretreated with 0.5 mM NAC for 30 min, the cells were treated with ((2,4,5-trifluorobenzyl)oxy)-benzene derivatives at 4 ㎍/㎖ for 48 h. The changes of MMP using JC-1 was observed by flow cytometry. ***p < 0.001, vs. control; ###p < 0.001, vs. TFOPM; ++p < 0.01, +++p < 0.001, vs. TFOBO alone. **(D)** After serum-starvation, THP1 cells were pretreated with 10 nM DPI for 30 min, followed by treatment with ((2,4,5-trifluorobenzyl)oxy)-benzene derivatives at 4 ㎍/㎖ for 48 h. The changes of MMP using JC1 was observed by flow cytometry. **p < 0.01, ***p < 0.001, vs. control; ##p < 0.01, ###p < 0.001, vs. TFOPM; ++p < 0.01, +++p < 0.001, vs. TFOBO alone. The graphs are presented as the mean ± SD (n = 3 replicates for each group). The representative data from three independent experiments are shown.

Uncropped gel image Figure 1D

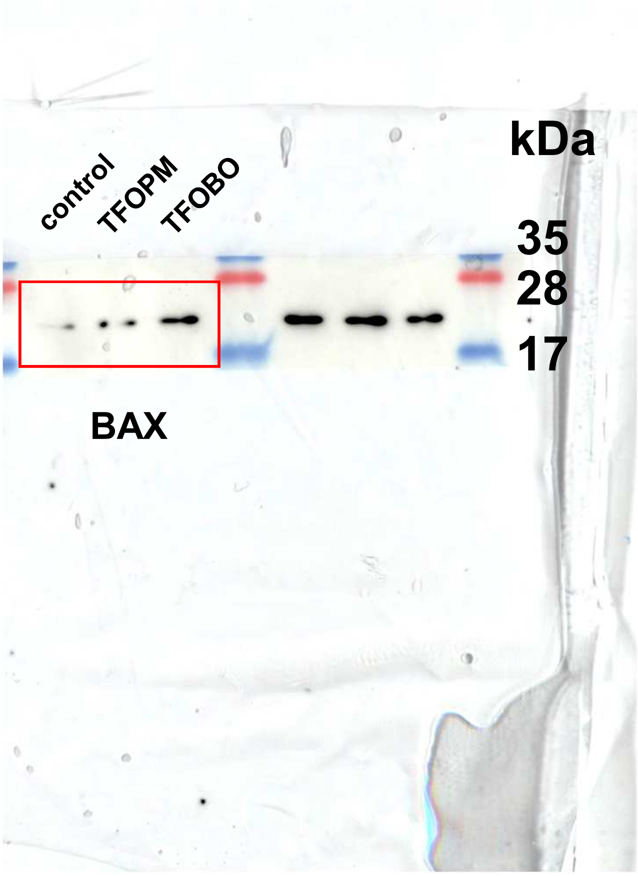

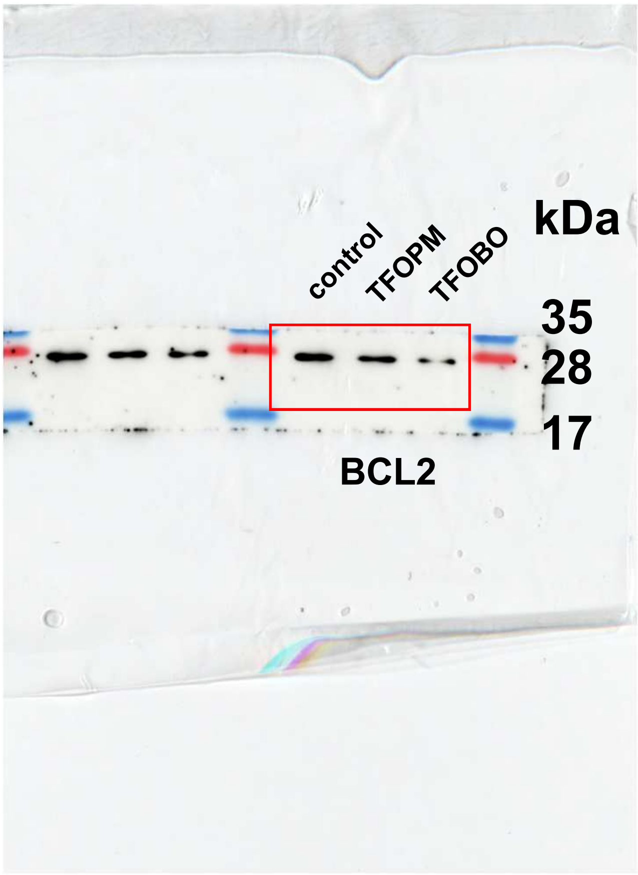

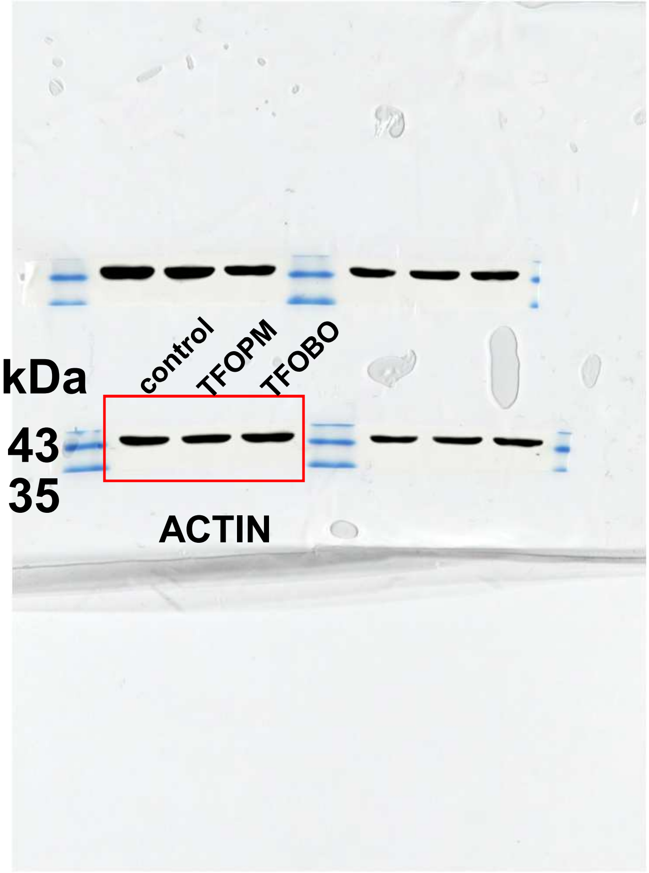

Uncropped gel image Supplementary Figure S1C

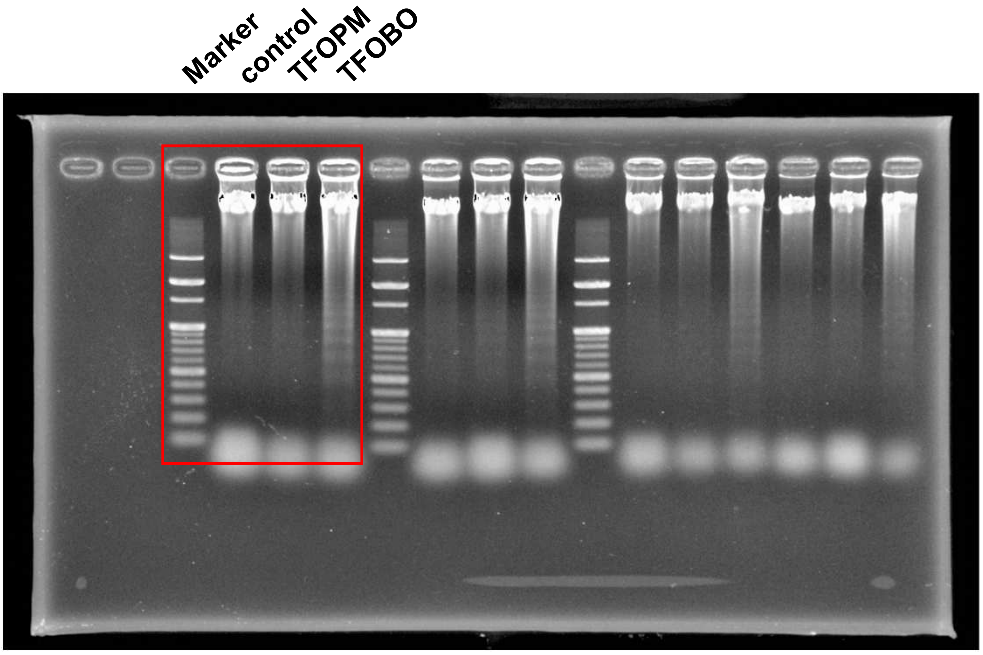

Supplement: Supplementary file 1 — Supplementary Figures. [file 41598_2022_11543_MOESM1_ESM.pdf]
